# Quantification of RPE Changes in Choroideremia Using a Photoshop-Based Method

**DOI:** 10.1167/tvst.9.7.21

**Published:** 2020-06-18

**Authors:** Yi Zhai, Manlong Xu, Ioannis S. Dimopoulos, David G. Birch, Paul S. Bernstein, Jenny Holt, David Kirn, Peter Francis, Ian M. MacDonald

**Affiliations:** 1 Department of Ophthalmology and Visual Sciences, University of Alberta, Edmonton, Alberta, Canada; 2 Department of Ophthalmology, University of Ottawa, Ottawa, Ontario, Canada; 3 Retina Foundation of the Southwest, Dallas, TX, USA; 4 Department of Ophthalmology and Visual Sciences, Moran Eye Center, University of Utah, Salt Lake City, UT, USA; 5 4D Molecular Therapeutics, Emeryville, CA, USA

**Keywords:** choroideremia, fundus autofluorescence, gene therapy

## Abstract

**Purpose:**

To develop a reliable and efficient method for quantifying the area of preserved retinal pigment epithelium (RPE), facilitating the evaluation of disease progression or response to therapy in choroideremia (CHM).

**Methods:**

The fundus autofluorescence images of CHM patients were captured at baseline and 1 year. A Photoshop-based method was developed to allow the reliable measurement of the RPE area. The results were compared with measurements generated by the Heidelberg Eye Explorer 2 (HEYEX2). The areas measured by two independent graders were compared to assess the test–retest reliability.

**Results:**

By using the Photoshop-based method, the area of the RPE measured from 64 eyes was seen to decrease significantly (*P* < 0.001) at a rate of 2.57 ± 3.22 mm^2^ annually, and a percentage of 8.39% ± 5.24%. The average standard deviations for Photoshop were less than that for HEYEX2 (0.5–1.1 in grader 1; 0.4–1.6 in grader 2), indicating less intragrader variability. The RPE decrease as determined by the Photoshop-based method showed excellent reliability with an intraclass correlation coefficient of 0.944 (95% confidence interval, 0.907–0.966). In Bland-Altman plots, the Photoshop method also exhibited better intergrader agreement.

**Conclusions:**

Photoshop-based quantification of preserved RPE area in patients with CHM is feasible and has better test–retest reliability compared with the HEYEX2 method.

**Translational Relevance:**

An accurate quantification method for longitudinal RPE change in CHM patients is an important tool for the evaluation of efficacy in any therapeutic trials.

## Introduction

Choroideremia (CHM; OMIM #303100) is an X-linked recessive retinal dystrophy that affects 1 in 50,000 to 100,000 individuals. It is caused by mutations in the *CHM* gene, which encodes Rab Escort Protein 1 (REP1).^1^ All mutations in *CHM* result in the truncation or absence of the normal protein product REP1, and this makes CHM a prime candidate for gene replacement therapy.^2^

Clinical trials of experimental therapies for inherited retinal therapies are challenging us to seek accurate outcome measures that will predict safety and efficacy. In previously reported trials of CHM gene therapy, ETDRS (Early Treatment Diabetic Retinopathy Study) letter acuity was used as a primary end point.^3^^–^^7^ As many CHM patients retain 20/20 visual acuity before age 40 years,^8^ using visual acuity as the primary outcome measurement would not fully reflect the efficacy of an intravitreal gene therapy targeting the whole retina. For that reason, we are interested in exploring outcome measures beyond visual acuity in CHM.

Fundus autofluorescence (FAF) is a noninvasive assessment providing in vivo information on retinal status. Blue or short-wavelength autofluorescence (BAF) imaging use a 486-nm laser light to excite lipofuscin, which is an endogenous fluorophore in retinal pigment epithelial (RPE) cells. As CHM causes degeneration of RPE,^9^ it typically presents with a distinctive FAF appearance. Classic FAF findings in CHM consist of an irregular, stellate-shaped central island of retained autofluorescence surrounded by an absent fluorescence background.^10^ In this report, we will provide a method to measure FAF area that could be adopted in these trials. Although we use the example of CHM, the method would have potential application in other RPE disorders, such as age-related macular degeneration, Stargardt disease, and others.

In recent studies, preserved FAF measured by Heidelberg Eye Explorer 2 (HEYEX2) version 1.10.0.0 (Heidelberg Engineering, Heidelberg, Germany) software was used to evaluate progression of CHM disease.^11^^,^^12^ Researchers simply used the “draw region” tool^12^ or the RegionFinder tool of HEYEX2^11^, which was designed to “provide a semi-automatic quantification of well-demarcated regions with significantly decreased autofluorescence signal intensity.” Nevertheless, as the preserved FAF area in CHM is usually irregular and not “well-demarcated,” HEYEX2 may not provide an accurate measurement.

In this study, we collected FAF data from a multicenter prospective natural history study of CHM to (1) establish a novel and reproducible method for quantifying the area of preserved RPE in CHM, and compare it with the HEYEX2-based protocol; (2) validate FAF area as a biomarker for disease progression in subjects with CHM; and (3) address grading challenges that may be encountered in the future CHM clinical trials.

## Methods

This study was a prospective, longitudinal, multicenter, observational study of CHM patients with a confirmed genetic diagnosis. All subjects were male patients, at least 14 years old, with visual acuity of 20/200 or better (corresponding to 34 ETDRS letters) for both eyes. Patients with concomitant retinal pathology and/or complicating systemic diseases were excluded from the study. All patients signed an informed consent form before being screened for the study. All patients underwent complete ocular examinations, fundus photography, visual field testing, and BAF imaging at the same visit. Visual acuity was recorded in each eye using the ETDRS charts, following standardized refraction. The protocol adhered to the tenets of the Declaration of Helsinki, was registered with ClinicalTrials.gov (NCT02994368), and approved by the University of Alberta human research ethics board.

Following pupil dilation, FAF images were captured using the Heidelberg Spectralis system (Heidelberg Engineering). The acquisition mode was either high-speed or high-resolution, based on the decision of the site's principal investigator. The Automatic Real Time setting was greater than 15 frames. Images were obtained using 486-nm light and a 30° or 50° field of view, centered on the fovea. The 30° FAF images were collected only if the investigator indicated that the total area of autofluorescence fell within the 30° field of view. The setting used for each study subject was constant over the study period to compare images longitudinally.

**Figure 1.**
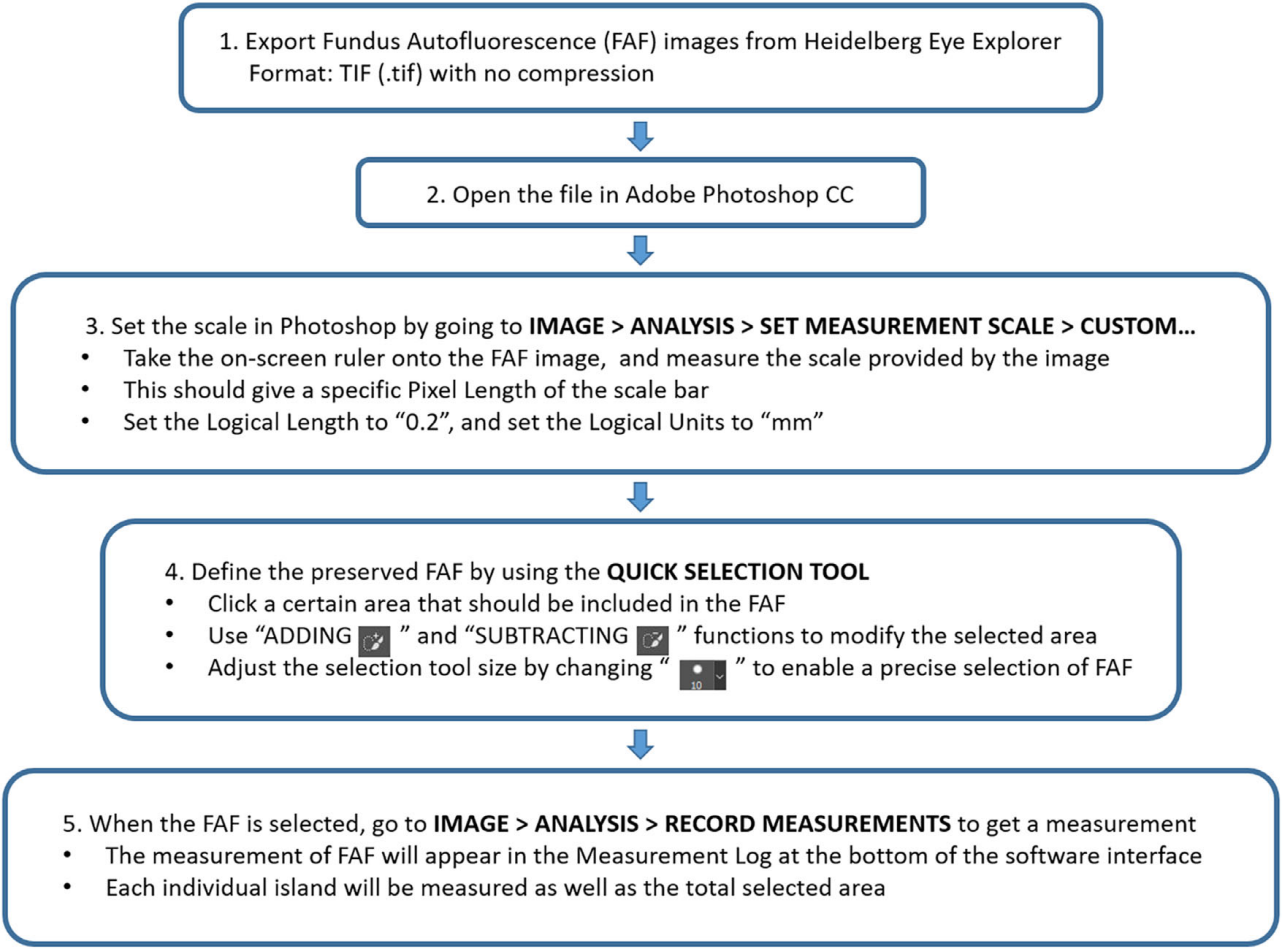
Preserved FAF Photoshop-based quantification methodology.

A novel method of quantifying preserved FAF in CHM using Photoshop CC software version 19 (Adobe Inc., Mountain View, CA) was developed (Fig. 1). We adjusted the size of the selection tool as needed and set the rest of the selection options as 100% hardness, 1% spacing, 100% roundness, and a 0° angle. Quantification results measured by HEYEX2 were used as controls. For the HEYEX2 protocol, we used the “draw region” function to facilitate the FAF measurements. Two clinical research fellows, both physicians, each with more than 2 years of experience testing patients in the Department of Ophthalmology and Visual Sciences, University of Alberta, worked as graders for this project. They were both trained in image acquisition and evaluation. Two graders (grader 1 as YZ and grader 2 as MX) independently defined the boundaries of preserved FAF areas on all the FAF images using both Photoshop CC and HEYEX2 protocols. Any preserved FAF island that measured greater than 0.1 mm^2^ was included in the analysis. Three measurements of preserved FAF using two methods were obtained in square millimeters (mm^2^).

As each image was measured three times by each grader, intragrader variability can be estimated by the variance within three measurements. The overall intragrader variability was calculated by taking an average of all the variances from the same methodology and then calculating the square root (average standard deviation [SD]). The mean of each group of measurements (three measurements for a single image done by one grader) was used for the further analysis.

The two methods were also compared graphically. The degree of intermethod agreement (concordance) was assessed according to the descriptive method of Bland and Altman.^13^

To further investigate the test–retest reliabilities of two preserved FAF quantification methods in a setting of future *CHM* gene therapy trials, intraclass correlation coefficients (ICC) of individual measurement and 1-year decrease measured by two graders were also calculated. Based on the 95% confidence interval (CI) of the ICC estimate, values less than 0.5, between 0.5 and 0.75, between 0.75 and 0.9, and greater than 0.9 indicate poor, moderate, good, and excellent reliability, respectively.^14^ A 2-way random-effects model was chosen to check the absolute agreement of the measurements generated by the two independent graders. A Bland-Altman plot was also used to evaluate the intergrader agreement of the two quantification methods.

Statistical analyses of the data were performed using IBM SPSS software version 26.0 (IBM Corp., Armonk, NY). Bland-Altman plots were generated by using GraphPad Prism version 8 for Windows 64-bit (GraphPad, San Diego, CA).

## Results

### Description of Study Data

Thirty-six CHM patients were recruited into this study from three sites. Images of four subjects were excluded from the analysis because of low image quality. The images from 64 eyes of 32 CHM patients were used in this study. The ages and best-corrected visual acuities (BCVA) of the study subjects are shown in Table 1. Each grader measured 128 images (64 from the baseline and 64 from 1-year visit) three times (i.e., 384 measurements per method) using two methods (HEYEX2 and Photoshop, i.e., 768 measurements). A total number of 1536 data points from two graders were included in this study.

**Table 1.** Baseline Characteristics

	
Subjects (eyes), *n*	32 (64)
Age in years, (mean ± SD)	35.03 ± 11.71
BCVA, letter score, (mean ± SD)	81.89 ± 9.01
>73, *n* (%)	57 (89.06)
34–73, *n* (%)	7 (10.93)

### General Comparison Between Two Methods

Preserved FAF areas quantified by both the Photoshop-based method and HEYEX2 software are shown in Table 2. To simulate a clinical trial setting, decreases in preserved FAF area over 1 year measured by two graders using two different methods were also calculated (Table 2). Measured with two different methods, the area of RPE in the central macula was seen to decrease significantly (*P* < 0.001) at a rate of 2.5 to 2.9 mm^2^ annually, and at a percentage of 8.1% to 8.9%. Considering an average of six measurements done by two graders using Photoshop, the area of RPE measured from 64 eyes was seen to decrease significantly (*P* < 0.001) at a rate of 2.57 ± 3.22 mm^2^ annually, and percentage of 8.39% ± 5.24%. The data measured by grader 1 using Photoshop were normalized (i.e., setting both the mean and SD of the data to 1) and plotted (Supplementary Fig. S1) to provide a general impression about the trend of FAF annual change in each study subject.

**Table 2.** Preserved FAF Areas and 1-Year Changes Measured by Two Independent Graders

	Grader 1		Grader 2	
	Photoshop	HEYEX2	Photoshop	HEYEX2
FAF area at baseline(mm^2^, mean ± SD, range)	30.3 ± 33.3(1.7 to 146.9)	27.4 ± 31.7(1.7 to 138.8)	30.0 ± 33.0(1.6 to 145.1)	27.5 ± 30.8(1.5 to 132.0)
FAF area at 1-year visit(mm^2^, mean ± SD, range)	27.7 ± 30.9(1.6 to 141.6)	25.1 ± 28.9(1.3 to 131.1)	27.1 ± 30.7(1.5 to 142.3)	24.6 ± 27.7(1.5 to 115.5)
One-year decrease(mm^2^, mean ± SD, range)	2.7 ± 3.3(−0.9 to 23.4)	2.8 ± 4.2(−0.3 to 14.9)	2.5 ± 3.2(−1.8 to 23.9)	2.9 ± 4.7(−0.4 to 13.6)
One-year decrease(%, mean ± SD%)	8.7 ± 5.4	8.5 ± 9.3	8.1 ± 5.4	8.9 ± 10.4

**Figure 2.**
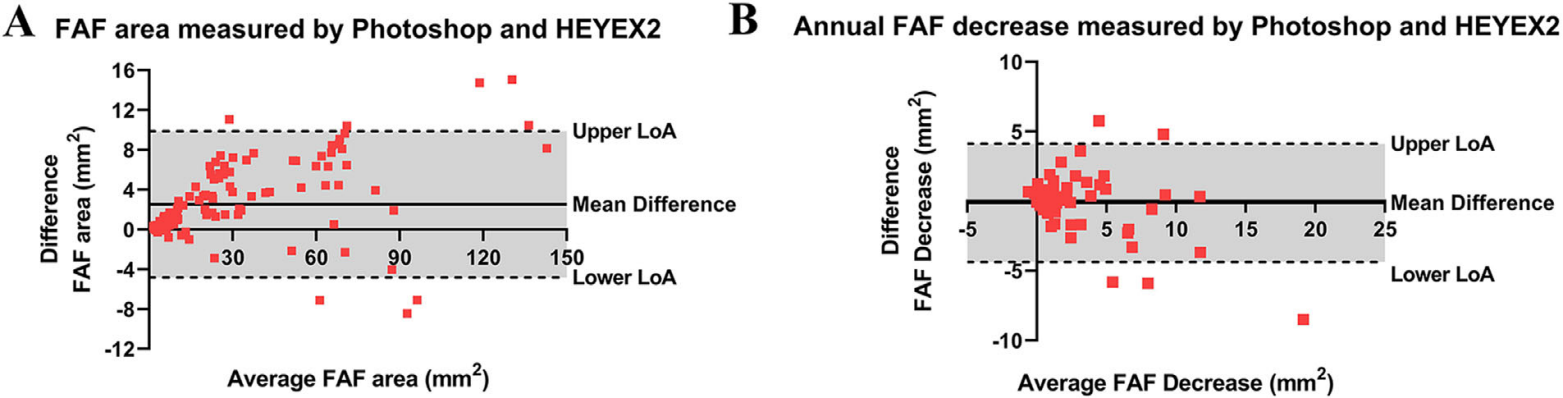
(A) Bland-Altman plot of preserved FAF area measured by two methods using data from grader 1. Difference (Photoshop minus HEYEX2) against the mean of each pair of measurements for the same image. *Solid line*: mean difference, 2.2 (95% CI, −5.0, 10.0), *top and bottom dashed lines*: upper and lower LoA (−4.8 to 9.8). (B) Bland-Altman plot of annual FAF decrease measured by two methods using data from grader 1. Difference (Photoshop minus HEYEX2) against mean of each pair of measurements for the same couple of images. *Solid line*: mean difference, 0.1 (95% CI, −4.2, 4.5), *top and bottom dashed lines*: upper and lower LoA (−4.4 to 4.1).

Data from grader 1 were used to evaluate the difference between two methods. A Bland-Altman plot (Fig. 2A) compared the average FAF area of all 128 measured pairs with the difference in measurements between the methods and revealed a 2.2 mm^2^ difference between two methods. Measurements using Photoshop were on average 2.2 mm^2^ (95% CI, −5.0, 10.0) greater that measurements by HEYEX2. The limits of agreement (LoA) ranged from −4.8 to 9.8. With the increase in average preserved FAF area, the intermethod agreement declined. Figure 2B plots the difference in FAF decreases against the mean of the FAF decreases measured by the two methods. The mean difference was 0.1 mm^2^ (95% CI, −4.2, 4.5), which indicates that the results measured by two methods are comparable in general. The LoA ranged from −4.4 to 4.1.

### Intragrader Variability for Two Methods

In terms of the intragrader variability (Table 3), the three measurements generated by the Photoshop-based method showed less average SDs in both graders compared with three measurements generated by HEYEX2, indicating the Photoshop-based method had less intragrader variability.

**Table 3.** Intragrader Variability of the Measurements

	Photoshop	HEYEX2
Intragrader variability grader 1(average SD)	0.5	1.1
Intragrader variability grader 2(average SD)	0.4	1.6

### Intergrader Agreement of Two Methods

The ICCs of the two quantification methods of preserved FAF is shown in Table 4. When measuring a single image, the ICC between the two graders were both considered to be excellent (1.000 for Photoshop and 0.997 for HEYEX2). Nevertheless, in terms of measuring the preserved FAF area change over 1 year, only the Photoshop-based protocol showed an ICC score that was considered to indicate excellent reliability (0.944, 95% CI, 0.907–0.966). The ICC score for the HEYEX2-based method was considered only as good reliability (0.819, 95% CI, 0.702–0.890).

**Table 4.** ICCs of Two Quantification Methods of Preserved FAF

	Photoshop	HEYEX2
ICC for individual measurements	1.000 (95% CI, 1.000–1.000)	0.997 (95% CI, 0.996–0.998)
ICC for 1-year decrease	0.944 (95% CI, 0.907–0.966)	0.819 (95% CI, 0.702–0.890)

Values <0.5, between 0.5 and 0.75, between 0.75 and 0.9, and >0.9 are indicative of poor, moderate, good, and excellent reliability, respectively.

**Figure 3.**
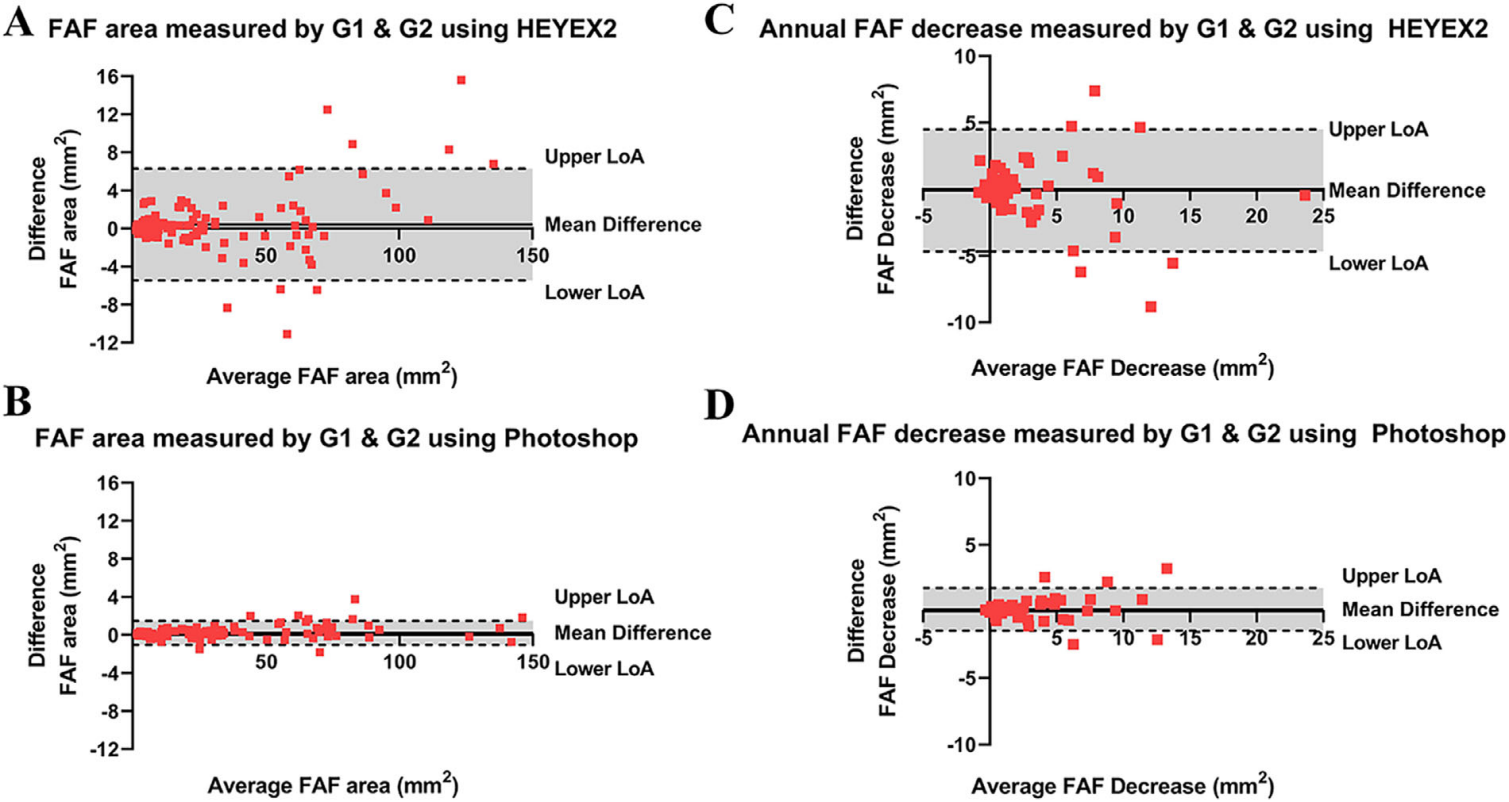
(A) Bland-Altman plot of preserved FAF area measured by two graders using HEYEX2. Difference (grader 1 minus grader 2) against the mean of each pair of measurements for the same image. *Solid line*: mean difference, 0.4 (95% CI, −5.6, 6.4), *top and bottom dashed lines*: upper and lower LoA (−5.5 to 6.3). (B) Bland-Altman plot of preserved FAF area measured by two graders using Photoshop. Difference (grader 1 minus grader 2) against the mean of each pair of measurements for the same image. *Solid line*: mean difference, 0.2 (95% CI, −1.1, 1.5), *top and bottom dashed lines*: upper and lower LoA (−1.0 to 1.5). (C) Bland-Altman plot of preserved FAF area decrease measured by two graders using HEYEX2. Difference (grader 1 minus grader 2) against the mean of each pair of measurements for the same image. *Solid line*: mean difference, −0.9 (95% CI, −5.5, 3.8), *top and bottom dashed lines*: upper and lower LoA (−4.7 to 4.5). (D) Bland-Altman plot of preserved FAF area decrease measured by two graders using Photoshop. Difference (grader 1 minus grader 2) against the mean of each pair of measurements for the same image. *Solid line*: mean = 0.1 (95% CI, −1.5, 1.8), *top and bottom dashed lines*: upper and LoA (−1.5 to 1.8). G1, grader 1; G2, grader 2.

Bland-Altman plots show that for preserved FAF quantified by the two methods (Figs. 3A, 3B), grader 1 tended to create a slightly greater measurement compared with grader 2 of 0.4 mm^2^ (95% CI, −5.6, 6.4) by HEYEX2, and 0.2 mm^2^ (95% CI, −1.1, 1.5) by Photoshop. The HEYEX2 method (Fig. 3A, LoA: −5.5 to 6.3) had greater intergrader variability compared with the Photoshop method (Fig. 3B, LoA: –1.0 to 1.5). In terms of intergrader comparison for annual FAF decrease, Bland-Altman plots reveal that the measurements generated by Photoshop had better intergrader agreement (mean difference, 0.1; 95% CI, −1.5, 1.8; LoA −1.5 to 1.8) compared with the measurements generated by HEYEX2 (mean difference, −0.9; 95% CI, −5.5, 3.8; LoA −4.7 to 4.5).

## Discussion

**Figure 4.**
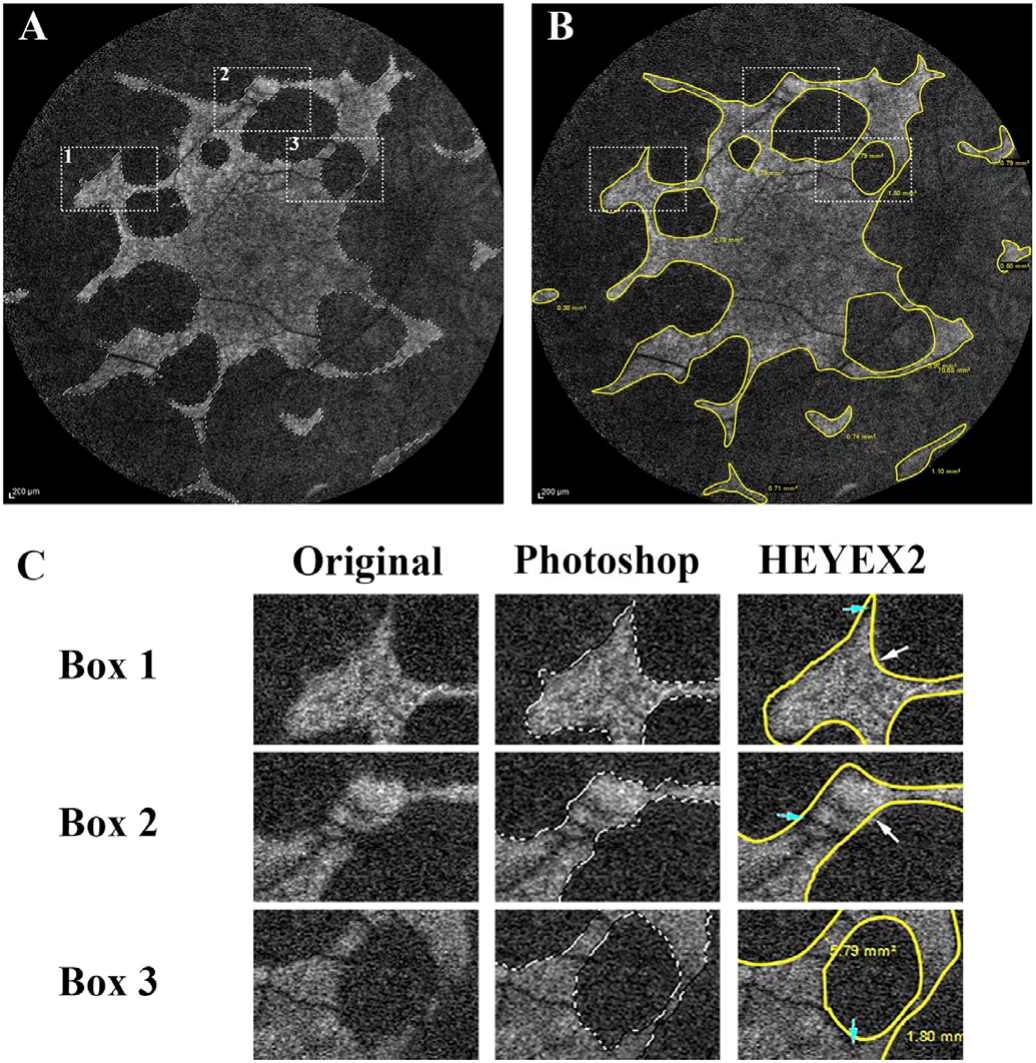
Examples of grading by Photoshop-based protocol and HEYEX2 protocol. (A) An example of grading by the Photoshop-based protocol; (B) an example of grading by the HEYEX2 protocol; (C) magnified images of box 1, 2, and 3. *White arrows* indicate the preserved FAF areas that were incorrectly excluded from HEYEX2 grading. *Cyan arrows* indicate the nonautofluorescent signal areas that were incorrectly included in HEYEX2 grading.

Photoshop is a powerful graphic editing software that has been previously used in medical imaging quantification.^15^ In the field of visual science, it has been used to quantify corneal vasculature change,^16^ as well as Goldmann visual fields.^17^ By using Photoshop in this study, we established a novel and reproducible method for quantifying the area of preserved RPE in CHM. We compared our protocol with the Heidelberg HEYEX2 protocol and found our protocol to be more reliable and less grader-dependent. From our experience, this difference was the result of two factors. First, the quick selection tool of the Photoshop software has powerful abilities to detect the intensity differences between pixels and does not require the selected regions to be smooth or continuous. This feature allows a better fit of the preserved FAF compared with HEYEX2 (Fig. 4), and can explain the findings that graders had greater disagreement in the cases with larger preserved FAF areas using HEYEX2. The images with greatest and least intergrader agreement are provided in the Supplementary material (Supplementary Fig. S2). Moreover, in cases with multiple preserved FAF islands, Photoshop software is able to directly generate a total area measurement after the grader finishes his or her selection. In contrast, the HEYEX2 protocol requires the graders to run additional calculations to get the total area measurement. This factor introduces one more calculation and may increase the chance of manual errors. In terms of measurement time, the Photoshop-based method takes a similar amount of time relative to the HEYEX2 protocol. Of note, we did not include the HEYEX2 RegionFinder tool in our comparison, as it can be more time-consuming compared with the other two methods.

Several trials of AAV2-REP1 gene therapy have been published, and all of them used change in BCVA as their primary end points.^3^^,^^5^^–^^7^ As all delivered the vector by subretinal injections and were designed to treat only the central retina, BCVA was a reasonable primary end point. However, some future gene therapy products in the pipeline aim to use an intravitreal approach to treat the whole retina (e.g., 4D-110; 4D Molecular Therapeutics, CA). The prospective patients to enroll in an intravitreal gene therapy trial will generally have mid-stage disease, and normal or close to normal BCVA. Therefore the ultimate goal for intravitreal gene therapy will be to maintain functional retina rather than to stabilize visual acuity. Consequently, surrogate biomarkers of CHM progression other than BCVA will be preferred in these trials. Preservation of RPE as measured by FAF area may be preferred in this setting. The novel quantification method of RPE presented in this study could be used in these future clinical trials, as it provides better intragrader and inter-reliabilities.

Our study has some limitations. For example, the precise measurement of FAF images in square millimeters also depends on other parameters, such as axial length and magnification. However, axial length would not affect the reported longitudinal measurements, as all pairs of eyes will be equally minified or magnified depending on their axial length. Secondly, Photoshop CC is not open source software, and users need to pay a monthly subscription fee. However, some universities may have a corporate rate for this subscription. A third relative limitation is that this protocol is most useful for prospective studies. It requires consistent FAF imaging settings (e.g., lens, A-scan setting, focus, etc.). Different FAF settings may create inaccuracies in scale, and eventually make the results less reliable. Also, one limitation of the FAF imaging itself is that it is only able to capture the preserved FAF within central 50°. Any remnant RPE beyond this field will not able to be detected by FAF imaging.

## Conclusions

We established a novel method to quantify preserved RPE area in CHM using Photoshop software. This method provides better inter- and intragrader reliabilities, especially for longitudinal RPE change measurement. This method will be a powerful tool for future intravitreal CHM gene therapy trials.

## Supplementary Material

Supplement 1
