# Supplementary material for: Quantification of RPE Changes in Choroideremia Using a Photoshop-Based Method
Source: Transl Vis Sci Technol. 2020 Jun 18;9(7):21. doi: 10.1167/tvst.9.7.21 (PMC7414628; doi:10.1167/tvst.9.7.21)
Supplement: Supplement 1 [file tvst-9-7-21_s001.pdf]

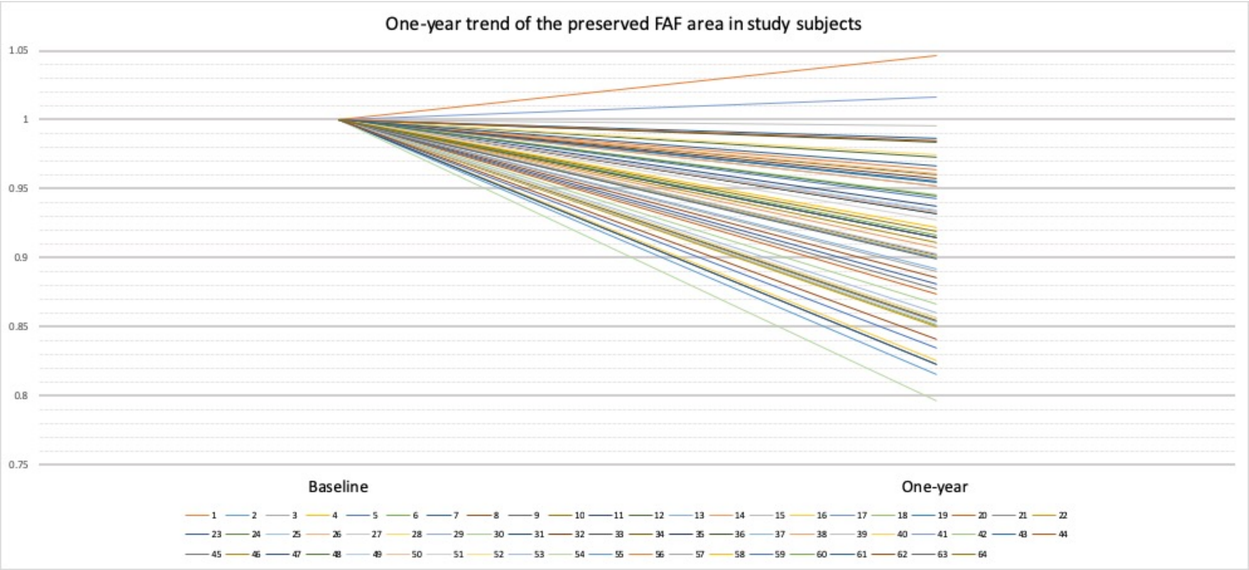

Figure S1. One-year changes in percentage of the preserved FAF area in study individuals.

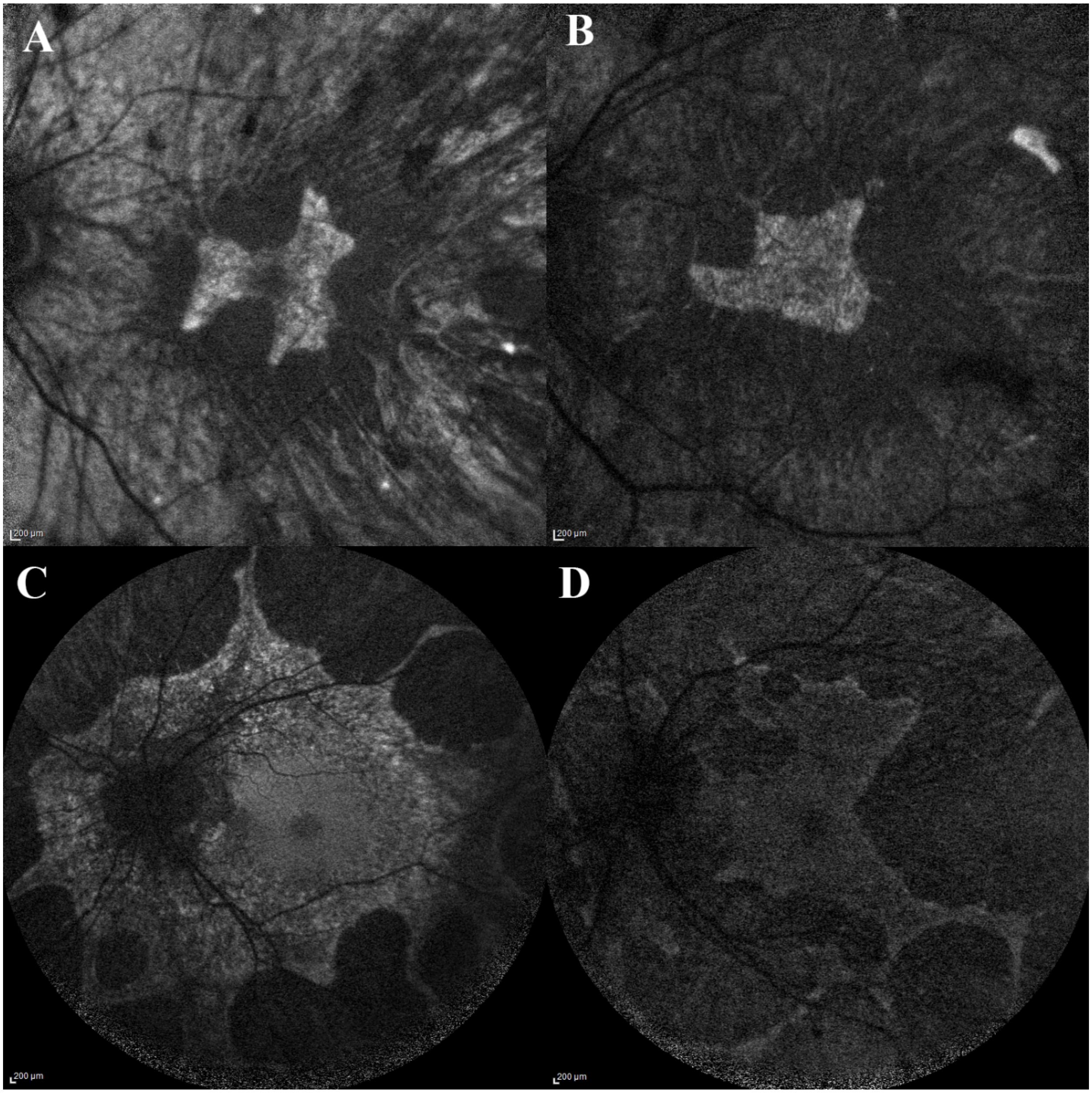

Figure S2. FAF images with greatest and least inter-grader disagreement. A) FAF image with least inter-grader disagreement using HEYEX2; B) FAF image with least inter-grader disagreement using Photoshop; C) FAF image with greatest inter-grader disagreement using HEYEX2; D) FAF image with greatest inter-grader disagreement using Photoshop.
